# Editorial: Testamentary capacity and undue influence in older adults

**DOI:** 10.3389/fpsyg.2024.1430893

**Published:** 2024-06-10

**Authors:** Christopher M. Nguyen, Stacey Lipio Brothers, Natalie L. Denburg

**Affiliations:** ^1^Department of Psychiatry and Behavioral Health, College of Medicine, The Ohio State University, Columbus, OH, United States; ^2^Department of Neurology, The University of Iowa, Iowa City, IA, United States

**Keywords:** testamentary capacity, aging, financial decision-making, neuropsychological assessment, forensic psychiatry

The projected growth of the population aged 65 and older globally marks one of history's most significant demographic shifts (United Nations, 2019). Within the United States, the number of older adults is projected to reach 21% of the population by 2030 (Mather et al., 2015). Consequently, clinicians providing healthcare and other services for older adults will increasingly be called upon to assess an individual's capacity to create or amend a will. Demand for services such as testamentary capacity (TC) evaluations is set to surge. Yet, despite the significant legal and financial implications, there is no standardized assessment procedure to determine TC. This Research Topic was proposed to invite review articles and original research investigating testamentary capacity. Four articles (Denburg et al.; Kenepp et al.; Roche; Shima et al.) were selected to provide a framework for TC evaluations, as well as insight into the current state of the field and considerations for practice.

The frameworks proposed by Roche and Kenepp et al. provide essential guidelines for testamentary capacity (TC) evaluations, offering detailed insights into the legal, clinical, and ethical aspects of assessing TC. In Roche's approach, a “four-bin” model (Behnke, 2014) incorporating legal, clinical, and ethical responsibilities, as well as risk management considerations, inherent in conducting a testamentary capacity (TC) evaluation was presented. Kenepp et al. advocated for a thorough examination of testamentary capacity (TC), emphasizing the necessity of assessing cognition, financial decision-making, testamentary knowledge, mood and psychiatric factors, and undue influence as integral components of the evaluation. Both Roche and Kenepp et al. reference the *Banks* vs. *Goodfellow* case of 1870, which set the current legal standard for TC, stating that individuals are assumed to be competent unless they meet any of the following criteria: (1) they do not understand what a will is, or the fact that they are making or changing a will; (2) they do not know their relationship to family members and/or other people whose interest may be affected by the will; (3) they do not understand the nature of their personal property; (4) they do not have a viable plan for the distribution of their property after death (Hoffman, 2018, p. 215). These are the criteria Roche encourages neuropsychologists to consider addressing the legal “bin” of the evaluation adequately, and Kenepp et al. considers part of the testamentary knowledge assessment.

From a clinical perspective, although there isn't a universally accepted set of tests for evaluating testamentary capacity (TC), Roche and Kenepp et al. both emphasize language, memory, and executive functioning as critical domains to assess. While both agree on the importance of assessing psychiatric factors that could impact TC, Kenepp et al. discuss how the *Banks* vs. *Goodfellow* case also established that having significant psychiatric concerns does not automatically negate one's TC. For example, if an individual presents with delusions, the clinician must assess whether and to what degree these delusions impact the four legal criteria mentioned above. Kenepp et al. also encourage clinicians to specifically assess financial decision-making, cautioning against self- and informant-reported measures, as they are prone to biases and cannot be verified (Ponsford et al., 2000; Martin et al., 2012; Sunderaraman et al., 2019). They subsequently suggest using performance-based tasks, though the literature is somewhat mixed on how well performance-based measures in a standardized environment can predict real-world functioning (Chaytor and Schmitter-Edgecombe, 2003). Additionally, it is notable that older adults tend to have the lowest levels of financial literacy across age groups (Lusardi et al., 2020), which could contribute to what others may perceive as poor financial decision-making when dividing assets, even in cognitively intact older adults. Denburg et al. conducted an experiment on healthy older adults to determine the effect of completing a self-evaluation worksheet based on information in legal documents related to a reverse mortgage (RM). They found that individuals who completed the self-evaluation worksheet better-comprehended RMs and were less interested in an RM. These findings suggest that encouraging older adults to interact with financial and/or legal information related to their assets may be one way in which to increase their comprehension, allowing them to make more informed decisions.

There are notable distinctions between the frameworks proposed by Roche and Kenepp et al.. Kenepp et al. discusses prior literature that suggests individuals should demonstrate intact cognitive functioning and an ability to make financial decisions objectively. This framework contrasts with Roche, who argues that a diagnosis of dementia or mild cognitive impairment, while indicative of impairment on testing, does not necessarily negate TC if the other criteria are met. Considering the fluctuating nature of TC (as noted by Kenepp et al.), clinicians must assess whether the diagnosis and current cognitive status are likely to remain stable or if there's potential for improvement where TC might be restored. For example, Roche presents a case vignette illustrating how psychotherapy to address significant mood symptoms could enhance an individual's ability to demonstrate capacity. Additionally, the findings of Denburg et al. suggest that increased exposure to financial and legal information may serve as another support for older adults to improve their comprehension and, thus potentially, their capacity.

The trajectory of TC evaluations appears poised for a significant evolution, driven by the impending surge in demand for services fueled by the aging demographic landscape of the United States. Building on the foundations laid by Roche and Kenepp et al., future directions in TC evaluations may involve refining and integrating these proposed frameworks to establish a comprehensive and standardized approach. Further advancements in clinical practice may entail a more nuanced evaluation of cognitive, psychiatric, and financial decision-making factors, as recommended by Kenepp et al.. Integration of performance-based tasks to mitigate biases inherent in subjective reporting, as suggested by previous research (Ponsford et al., 2000; Martin et al., 2012; Sunderaraman et al., 2019), could enhance the accuracy and reliability of TC assessments.

The imperative for standardization extends beyond the realm of neuropsychology, as highlighted by Shima et al. in geriatric forensic psychiatry, emphasizing the interdisciplinary nature of TC evaluations. Collaborative efforts among legal, clinical, and ethical stakeholders will be essential in establishing and implementing standardized procedures to ensure equitable and robust TC assessments amidst the escalating demand for services. Moreover, future research endeavors might explore innovative approaches to enhance older adults' comprehension of financial and legal information, as proposed by Denburg et al.. Addressing existing discrepancies between frameworks, particularly concerning the impact of cognitive impairment on TC and the potential for capacity improvement over time, will remain critical focus areas.

In conclusion, embracing a holistic approach encompassing legal, clinical, and ethical dimensions will be pivotal in navigating the complexities of TC evaluations in the future. This comprehensive approach holds the promise of more accurate and reliable assessments, meeting the growing needs of older adults in society. The articles presented in this Research Topic and examined in this editorial can serve as foundational resources, guiding clinicians and researchers in addressing the evolving landscape of TC assessments and meeting the growing needs of older adults in society.

## Author contributions

CN: Conceptualization, Formal analysis, Investigation, Methodology, Project administration, Supervision, Writing – original draft, Writing – review & editing. SB: Writing – original draft, Writing – review & editing. ND: Writing – original draft, Writing – review & editing.
